# Effect of mixed planting on soil nutrient availability and microbial diversity in the rhizosphere of *Parashorea chinensis* plantations

**DOI:** 10.3389/fmicb.2024.1464271

**Published:** 2024-10-15

**Authors:** Wannian Li, Le Xie, Yuanyuan Xu, Mei Yang

**Affiliations:** ^1^Key Laboratory of National Forestry and Grassland Administration on Cultivation of Fast-Growing Timber in Central South China, College of Forestry, Guangxi University, Nanning, China; ^2^Guangxi Colleges and Universities Key Laboratory for Cultivation and Utilization of Subtropical Forest Plantation, Guangxi Key Laboratory of Forest Ecology and Conservation, College of Forestry, Guangxi University, Nanning, China; ^3^Forestry Seedling Station of Guangxi, Nanning, China

**Keywords:** Dipterocarpaceae, mixed planting patterns, rhizosphere soil, nutrient bioavailability, microbiological diversity

## Abstract

**Introduction:**

*Parashorea chinensis* Wang Hsie (*Pc*) is an endangered tree species endemic to tropical and subtropical China. However, the acidic red soil areas where it is distributed generally face nutrient limitation. The study of the effects of mixed planting on soil biogeochemical processes contributes to the sustainable management and conservation of *Pc*.

**Methods:**

We selected pure and mixed stands of *Pc* and collected its rhizosphere and bulk soil samples to clarify the effect of mixed planting on the soil microbial community and the nutrient status.

**Results:**

The results showed that (1) All stands were strongly acidic phosphorus-deficient soils (pH < 4.0, available phosphorus <10.0 mg·kg^−1^). There was a significant rhizosphere aggregation effect for soil organic C, total and available N and K, microbial biomass, and inorganic P fraction. (2) The mixed planting significantly increased the soil water content, organic C, available nutrients, the activities of β-1,4-glucosidase and urease, and microbial biomass. The inorganic P fractions are more influenced by rhizosphere, while organic P fractions are more influenced by tree species composition. (3) Fungi and their ecological functions are more susceptible to tree species than bacteria are, and have higher community compositional complexity and α-diversity in mixed plantations. And mixed planting can improve network complexity among key microorganisms. (4) The correlation between soil microorganisms and environmental factors was significantly higher in mixed forests than in pure forests. Soil organic C, available N and P, microbial biomass C and N, β-1,4-glucosidase, and stable P fractions were the key environmental factors driving changes in fungal and bacterial communities.

**Conclusion:**

In conclusion, the mixed planting patterns are more advantageous than pure plantations in improving soil physicochemical properties, enhancing nutrient effectiveness, and promoting microbial activities and diversity, especially *Pc* mixed with *Eucalyptus grandis × E. urophylla* is more conducive to soil improvement and sustainable management, which provides practical references for relocation protection of endangered tree species and species selection and soil fertility management in mixed planting. In addition, the study highlighted the key role of rhizosphere microenvironment in soil nutrient cycling and microbial community structure, which provides new perspectives for a deeper understanding of soil-microbe-plant interaction mechanisms.

## 1 Introduction

Soil microorganisms play a key role in nutrient cycling through the decomposition and mineralization of organic matter and the release and transformation of inorganic matter (Fierer et al., 2021). The establishment of mixed forests not only improves the diversity and composition of understorey vegetation and soil microbial communities, but also improves soil physicochemical properties and microbial functional and catabolic diversity (Gillespie et al., 2021). As tree diversity increases, the complexity of soil microbial biomass, plant-microbe interactions also increases (Chen et al., 2019). Not only does the symbiotic network of soil microbiota become more complex during the transition from pure to mixed forests, but nutrient limitation shifts from carbon (C) to nitrogen (N) and phosphorus (P) limitation. Therefore, while focusing on the effects of mixed silviculture on soil microbial communities, nutrient bioavailability and limitation characteristics, as well as on the relationship between soil microbes and nutrient availability, cannot be ignored.

Acidic soils are less effective than other soil types for P and potassium (K), leading to soil nutrient limitation, which has become an urgent challenge in agroforestry production (Zhu et al., 2018; Xia et al., 2022). Subtropical red soils are widely distributed in southern China, which are typically characterized by low pH and rich in clay minerals and iron (Fe) and aluminum (Al) oxides. However, these clay minerals, oxides of Fe^3+^, Al^3+^ and Ca^2+^, and hydroxides combine with most of the phosphates in the soil to become ineffective P, resulting in P-limited soils (Li et al., 2024). In addition, soil acidity reduces the availability of essential nutrients, such as calcium (Ca) and magnesium (Mg), and can also lead to toxic effects of Al and Mn on the plant root system, resulting in a significant reduction in plant growth and development (Huang et al., 2023). In the hilly red soil regions of southern China, the accumulation of plantation forest apoplastic litter also leads to soil acidification and nutrient limitation such as N and P (Zhan et al., 2009; Jin et al., 2023). Mixed afforestation strategies are significantly effective in alleviating nutrient limitation, confirming that there is a strong link between nutrient bioavailability and tree species (Yang et al., 2024). Mixed forests alter soil microbial functioning by affecting nutrient availability and root resource acquisition in forest apoplasts, which is driven by tree species composition (Dawud et al., 2017). Especially phosphorus limitation may play an important role in determining the stability of soil microbial symbiotic networks during tree species diversification (Gao et al., 2024). With the restoration of subtropical broadleaf mixed forests, soil nutrient limitation also shifted from N limitation to P limitation, which further emphasizes the critical role of P in forest restoration (Yang et al., 2021). Tree-mix silviculture may affect the structure of P fractions (Marschner et al., 2011). However, there is limited research on rhizosphere microbes in relation to P fractions and P effectiveness in mixed forests on acidic soils (Maharjan et al., 2018). Soils are spatially heterogeneous, and many soil conditions can vary dramatically with space (Fierer et al., 2021). The rhizosphere is the most active region of the soil microbial community, is more capable of breaking down the available substrate, and the structure of the community is different from that of bulk soils (Yu et al., 2018). Bulk and rhizosphere soils at different depths undergo different adaptation processes (Herre et al., 2022). Mixing effects were inconsistent across sampling locations and soil horizons, with differentiation and complementarity of ecological niches among tree species having greater advantages than pure forests for increasing soil nutrient content and effectiveness (Dawud et al., 2017). It is crucial to study the impact of mixed planting on rhizosphere and bulk soil fertility indicators and microbial diversity, which is useful to better understand the ecological function of rhizosphere effects in nutrient activation and microbial building processes.

*Parashorea chinensis* Wang Hsie (*Pc*) is a canopy species of the Dipterocarpaceae family endemic to China and the emblematic species of tropical seasonal rainforests (Jin et al., 2024). Its distribution area is indicative of the delineation of tropical forest vegetation types, and is also important for the conservation and study of endangered species of the Dipterocarpaceae family (Wang et al., 2024). However, it is facing many threats to its survival, such as its need for high temperature and humidity for growth, as well as alternating wet and dry frost-free environments (Ullah et al., 2024). Its native communities have narrow and fragmented distribution areas, low seed production and extensive fruit drop due to seed recalcitrance, and high seedling mortality making natural regeneration difficult (Su et al., 2014). Inadequate root development, insufficient nutrient uptake dynamics and growth retardation have been found in artificial seedlings and silviculture (Kaewgrajang et al., 2014; Jin et al., 2015; Dossa et al., 2019; Duan et al., 2019). In addition, it was once over-harvested and utilized due to its excellent timber attributes and high economic value, reasons that ultimately led to it being listed as an endangered species by the International Union for Conservation of Nature and Natural Resources (IUCN) and protected at the highest level by the Chinese government (Qin et al., 2017; Ming, 2021). Therefore, by studying the growth characteristics and environmental features of *Pc*, high-quality artificial cultivation and *ex situ* conservation are the best options for expanding its community numbers and restoring its habitat (Li et al., 2005). The results of previous studies have shown that severe P and N limitation in soils of *Pc* pure plantation, which may be responsible for its endangerment (Li et al., 2023); whereas mixed plantations of *Pc* are with better for the maintenance of soil nutrients and microbial functions, and mixed forests are an effective way to save endangered species of Dipterocarpaceae (Li et al., 2022). However, there is a lack of research on how mixed forests affect the spatial variation of soil microbial communities and the relationship between the availability of N, P, K and other nutrients (Waring et al., 2013; Zhang et al., 2021). For these reasons, we chose pure and mixed plantations of *Pc* as the subjects of our study. We hypothesized that mixed planting would be more conducive to increasing soil microbial diversity and nutrient availability, and thus alleviating nutrient limitation, compared to pure stands. And will address the following questions: (1) Does tree-species mixing enhance the rhizosphere aggregation and alter vertical spatial distribution characteristics of soil nutrients and microorganisms? (2) Does mixed forestry enhance soil fertility and activity and alleviate nutrient limitation faced by acidic red soils? (3) What are the key environmental variables that we are trying to explain the changes in microbial diversity in the rhizosphere driven by the mixing effect? This study aims to verify the positive role of mixed planting in alleviating nutrient limitation and enhancing microbial activity and diversity in the acidic red soil area. It also reveals the core factors driving changes in the rhizosphere effect of microorganisms, and ultimately provides practical guidance for the artificial management and relocation conservation of endangered tree species in the acidic red soil region.

## 2 Materials and methods

### 2.1 Experimental site

The experimental sample site of this study was located in the subtropical region of China, within the arboretum area of Nanning City, Guangxi Zhuang Autonomous Region (22°37′57″N, 108°18′47″E), and a detailed stand overview and geographical position is shown in Table 1 and Supplementary Figure S1. In January 2012, pure stands (denoted as PP) of *Parashorea chinensis* (*Pc*), mixed stands (denoted as MPE) of *Pc* and *Eucalyptus grandis × E. urophylla*, and mixed stands (denoted as MPD) of *Pc* and *Dalbergia odorifera* were established. As shown in Table 2, these plantation stands had similar soil parent material and properties, as well as close nutrient content at the time of establishment; the soil types were all dominated by red soil, which corresponds to laterite in the soil classification system of the Food and Agriculture Organization of the United Nations (FAO-UNESCO). In addition, these stands have similar climatic and topographic conditions, as shown in Supplementary Figure S1, they were all situated south of the Tropic of Cancer (23°26′N) and have a southern subtropical monsoon climate with an average annual temperature of 21°C, an average annual precipitation of about 1,300 mm. When selecting pure and mixed experimental stands, in addition to considering the above environmental factors to be as consistent as possible, it was also ensured that they had the same stand density, fertilizer standards and forest tending operations. In this way, data inaccuracies due to spatial heterogeneity will be avoided. Specifically, all stands were initially planted at a density of about 1,665 trees·ha^−1^, and a base fertilizer was applied to each tree at the time of planting. In the first 2 years after planting, a follow-up fertilizer was applied in May each year.

**Table 1:** Sample plot profiles of plantations with different planting patterns.

Planting patterns	Species composition	Age of stand	Management density (plants·ha^−1^)	Tree height (m)	Diameter at breast height (cm)	Average crown width (m)	Stand canopy density	Altitude (m)	Aspect of slope	Position of slope	Slope (°)
			Parashorea chinensis/mixed species								
PP	*Parashorea chinensis*	8	1,597	9.1	6.0	4.1	0.5	171.2	Southeast	Middle	20
MPE	*Parashorea chinensis × (Eucalyptus grandis × E. urophylla)*	8	1,232/411	9.3/19.6	6.1/15.7	3.9/4.3	0.6	178.9	Southeast	Middle	19
MPD	*Parashorea chinensis × Dalbergia odorifera*	8	904/706	9.0/7.5	5.9/7.8	3.2/3.6	0.6	176.1	Southeast	Middle	17

**Table 2:** Basic properties of the soil at the time of afforestation in 2012.

Planting patterns	Species composition	TN (g·kg^−1^)	TP (g·kg^−1^)	TK (g·kg^−1^)	AN (mg·kg^−1^)	NN (mg·kg^−1^)	AP (mg·kg^−1^)	AK (mg·kg^−1^)
PP	*Parashorea chinensis*	0.54	0.32	4.25	1.29	3.99	0.66	45.18
MPE	*Parashorea chinensis × (Eucalyptus grandis × E. urophylla)*	0.59	0.27	4.20	1.34	3.31	0.63	41.73
MPD	*Parashorea chinensis × Dalbergia odorifera*	0.6	0.31	4.62	1.26	4.11	0.62	43.88

Samples for the data sources in the table were collected from bulk soils from 0 to 20 cm depth. Collected and measured in 2012, each planting pattern contains three replicate samples. TN, total nitrogen; TP, total phosphorus; TK, total potassium; AN, ammonium nitrogen; NN, nitrate nitrogen; AP, available phosphorus; AK, available potassium.

### 2.2 Experimental design and sample collection

In July 2020, stands of three planting patterns (PP, MPE and MPD) were selected within the above study area. Three standard sample plots of 400 m^2^ (20 m × 20 m) were set up within each planting pattern, with plots spaced at least 200 m apart from each other. Within each standard sample plot, five *Pc* plants with close growth (their diameter at breast height (DBH) was close to the average DBH of the entire stand) were randomly selected. The area of their root distribution was used as the collection site for rhizosphere soil samples, and another five sites were selected as bulk soil sampling sites in areas with no root distribution evenly distributed within the sample plots. When collecting soil samples, the following scientific methods and steps were followed to ensure that the samples were sufficiently representative and to minimize bias: firstly, the root distribution area of each sampled target tree was identified, which are usually the parts of the tree where the root system is the most dense and the soil-root interactions are the strongest, and are therefore the optimal locations for rhizosphere soil collection. Secondly, a reasonable sampling depth is determined, and since the top soil layer may be disturbed by external factors, it is necessary to remove the top soil layer before sampling. After sampling, mixing and splitting are carried out to ensure homogeneity and representativeness of the samples. Aseptic tools are used throughout the sampling process to ensure that the soil is not contaminated. The method is as follows: after removing about 5 cm of top soil, the fine roots were excavated (to a vertical depth of 40 cm or less) in the direction of the root extension, and soil adhering to the fine roots (5 mm from the root surface) was collected with a brush as rhizosphere soil sample (Clausing et al., 2021). When collecting bulk soil samples, the surface apoplastic material was firstly removed, and the soil was collected vertically downwards in the area without plant roots in the depth ranges of 0–20 cm and 20–40 cm, respectively. Finally, the samples from all collection points of each sample plot were mixed into one soil sample and divided into three parts. The first partition was air-dried and sieved (pore size 0.15 mm) for the determination of soil physicochemical properties, nutrient content and phosphorus fractions, while the second partition was refrigerated in fresh state (4°C) for the determination of soil microbial biomass and extracellular enzyme activities. The third was frozen at ultra-low temperature (−80°C) for high-throughput sequencing of fungi and bacteria (Li et al., 2023).

### 2.3 Determination of soil physicochemical properties, nutrient content and phosphorus forms

For the determination of electrical conductivity (EC), a conductivity meter was used at 25°C after leaching the soil (10 g) with distilled water (50 mL); Soil moisture content (WC) was determined by weighing method. The pH was determined using a pH meter after leaching the soil (10 g) using distilled water (25 mL); Determination of organic carbon (SOC) by the K_2_Cr_2_O_7_-H_2_SO_4_ mixed heating oxidation-colorimetric method using a Titrette titrator; total nitrogen (TN) was determined by H_2_SO_4_-catalyst digestion using a continuous flow chemistry analyzer type AA3. Total phosphorus (TP) and total potassium (TK) were determined using inductively coupled plasma mass spectrometry with the NaOH melting method (Bao, 2000). Available phosphorus (AP) was determined by filtration using a double acid leaching method using an ultraviolet spectrophotometer, Available potassium (AK) was determined using flame photometer. Ammonium nitrogen (AN) and nitrate nitrogen (NN) by KCl leaching using a continuous flow analyzer type AA3 (Kammann et al., 2015). Soil β-1,4-glucosidase activity (Glu) activity was determined using p-nitrophenyl-β-glucopyranoside (C_12_H_15_NO_8_) as a substrate at 410 nm with a spectrophotometer at 410 nm; Urease activity (Ure) was determined by the Sodium phenol hypochlorite colorimetric method using urea (CO(NH_2_)_2_) as substrate; Acid phosphatase activity (Acp) by colorimetric method using sodium benzene phosphate (C_6_H_9_Na_2_O_6_P) as a substrate (Guan, 1986). To determine the effective aluminum (Al), a desilication leach solution at pH 7.3 was used with the KCl substitution-EDTA volumetric method. The effective iron (Fe) was measured by atomic absorption spectrophotometry (AAS method) using an acetylene-air flame.

Soil available nitrogen, ANN:


(1) ANN=Ammonium nitrogen+nitrate nitrogen

Soil microbial biomass carbon, nitrogen, and phosphorus (MBC, MBN, and MBP) (Equations 2–4) were determined by chloroform fumigation and leaching, and were calculated based on the difference between the measured contents of fumigated and unfumigated soil (*Ept*) and the conversion factor (*Kp*):


(2) MBC=EC/Kc

(3) MBN=EN/Kc

(4) MBP=Ept/Kp

Where EC, EN and *Ept* are the difference between the amount of organic C, total N and organic P in the leachate of fumigated and unfumigated soil samples, respectively, and *Kc* and *Kp* denote the conversion factor of 0.45 and 0.4, respectively (Brookes et al., 1985; Yu et al., 2011; Fanin et al., 2013).

Bioavailability of nitrogen, BN:


(5) $$BN=\left(\mathrm{Ammonium nitrogen}+\mathrm{nitrate nitrogen}\right)/\left(TN*1000\right)*100\%$$

Bioavailability of phosphorus, BP (Chen et al., 2020):


(6) $$BP=AP/\left(TP*1000\right)*100\%$$

Bioavailability of potassium, BK:


(7) $$BK=AK/\left(TK*1000\right)*100\%$$

Soil P was fractionated according to the method of Hedley et al. (1982) and He et al. (2021), which classified it into four inorganic and four organic P forms sequentially. The fractionation of the four inorganic P forms was determined according to the Chang-Jackson method (Chang and Jackson, 1957), which included Aluminum-bound P (Al-P), Iron-bound P (Fe-P), Calcium-bound P (Ca-P) and Occluded P (O-P). The four organic P forms was determined according to the Bowman-Cole method (Bowman and Cole, 1978), which included labile organic P (LOP), moderately labile organic P (MLOP), moderately resistant organic P (MROP), and highly resistant organic P (HROP).

### 2.4 High throughput sequencing of soil microorganisms

Rhizosphere soil samples were subjected to high throughput sequencing by Novegene Technology Co. DNA purity and concentration were assessed using agarose gel electrophoresis. Diluted genomic DNA was used as a template for PCR with specific primers containing barcodes. The bacterial diversity was determined using 16S V4 region primers (515F, 5′-GTGCCAGCMGCCGCGGTAA-3′ and 806R, 5′-GGACTACHVGGGTWTCTAAT-3′), while fungal diversity was determined using ITS1 region primers (ITS5-1737F and ITS2-2043R). The PCR products were examined by electrophoresis, and the target bands were recovered. Library construction was performed using the TruSeq® DNA PCR-Free Sample Preparation Kit. Sequencing was conducted on the Illumina NovaSeq 6000. Sequences with 97% similarity were clustered into operational taxonomic units (OTUs) and annotated with species classification.

### 2.5 Statistical analysis of data

Initial data collection and statistics on soil environmental factors were performed using Microsoft Excel 2019. Outliers were measured by standard deviation and the effect of outliers significantly deviating from the data center was reduced by logarithmic transformation. The Shapiro–Wilk test was used to verify the statistical distribution, and the values of the non-normal distribution were logarithmically converted to 10 (Li et al., 2023). The One-way ANOVA and Three-way ANOVA were conducted using SPSS (IBM SPSS Statistics 25.0) for each of the environmental indicators in this study. To determine whether the three categorical variables (planting pattern [PP, MPE, MPD], soil type [rhizosphere soil, bulk soil], soil layer [0–20 cm, 20–40 cm]) had a significant effect on the results and whether there was an interaction effect between any two categorical variables. Principal component analyses were performed on these indicators using Origin 2023. Mantel test analyses were performed using the “Vegan” and “linkET” packages of R software (Version 2.15.3) for plotting, including LEfSe (LDA Effect Size) analysis and cluster analysis for fungi and bacteria, RDA (Redundancy Analysis) for environmental factors and microbial communities, and NMDS (Non-metric multidimensional scaling) analysis based on Unweighted Unifrac. Significance levels were all set at *p* < 0.05 and *p* < 0.01. After filtering out OTUs with mean relative abundance less than 0.01% and less than 1/5 of the total sample size using R software, microbial co-occurrence network metrics were computed using the WGCNA package, and the network was constructed to present the community co-occurrence relationships of microorganisms. The OTUs that appeared in at least half of the samples in the corresponding group were retained for network analysis (Bai et al., 2023). The networks were established by calculating correlations among OTUs in different planting patterns, and the co-occurrence patterns were explored based on strong (spearman’s (|*r*|) > 0.6) and significant correlations (*p*-values <0.001). The networks were visualized using the interactive platform Gephi v. 0.9.2 (Gong et al., 2023).

## 3 Results and analysis

### 3.1 Soil physicochemical properties and nutrient bioavailability

**Figure 1:**
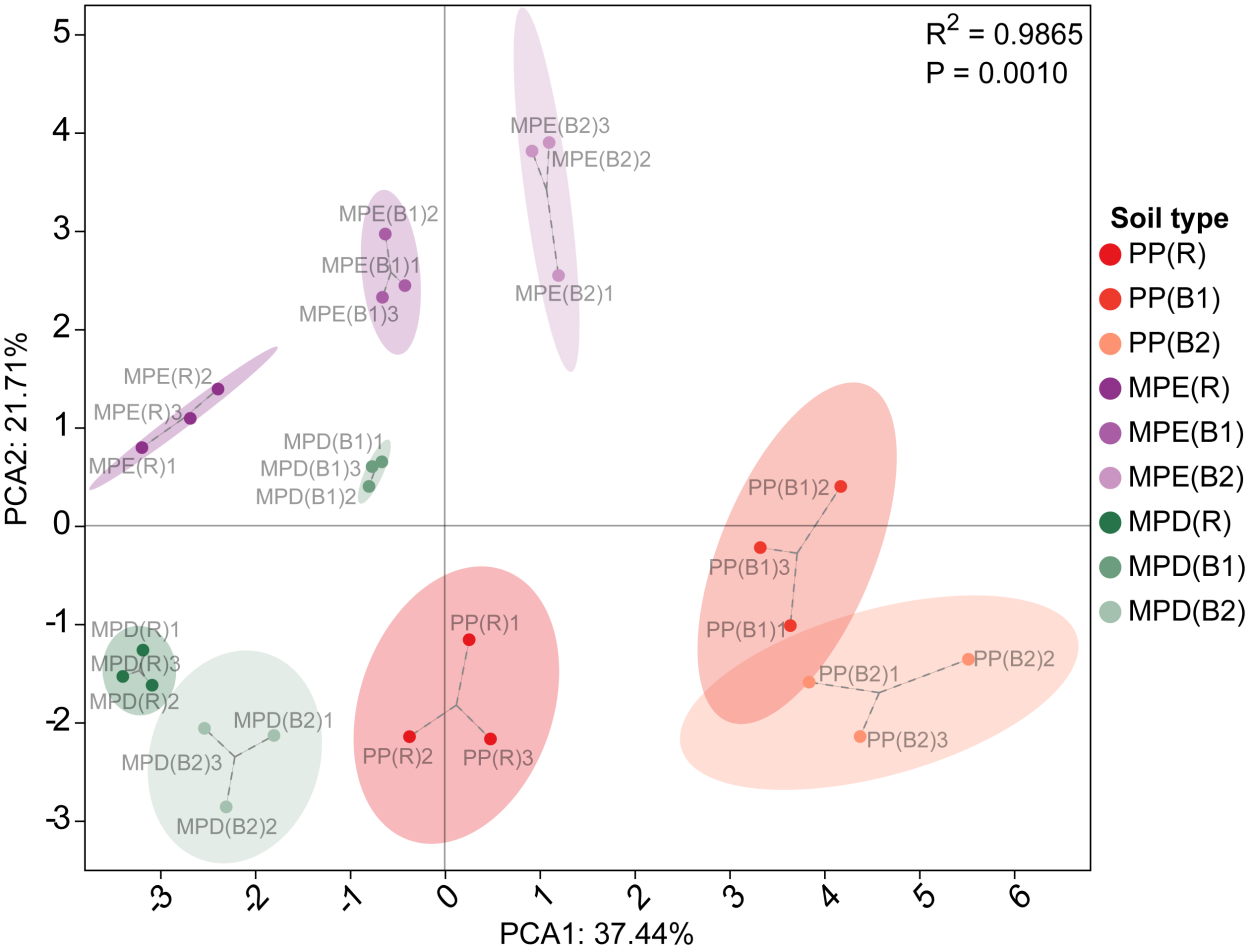
Principal component analysis of soil physicochemical properties and nutrients. PP, pure *Parashorea chinensis* plantations; MPE, *P. chinensis* trees in the mixed plantations of *P. chinensis* and *Eucalyptus grandis × E. urophylla*; MPD, *P. chinensis* trees in the mixed plantations of *P. chinensis* and *Dalbergia odorifera*. R, rhizosphere soil; B1, bulk soil (0–20 cm); B2, bulk soil (20–40 cm). In this study, the principal component results were ranked in descending order of variance, with PC1 having the largest variance and explaining the largest variation in the data, and PC2 having the next largest variance and further explaining the remaining variation. The variance contribution of PC1 was the highest, and that of PC2 the second highest. The three environment variables with the largest absolute values of load for PC1 are SOM, SOC and TN, and for PC2 are TP, AK and ANN.

The pH of all stands’ soils was less than 4, indicating that the local soils were strongly acidic. Mixed stands have the potential to enhance soil fertility, soil organic carbon (SOC), total and available N and K, and the bioavailability of phosphorus (P) are higher than in pure stands (Table 3). They were highly significantly affected by tree species composition (*R*^2^ = 0.9865, *p* = 0.001), with PP and MPE in particular showing the greatest differences (Figure 1). In terms of rhizosphere effects, exchangeable Fe and Al were significantly higher (*p* < 0.05) in rhizosphere than in bulk soils in pure plantations, which may indicate potential metal toxicity risks. In mixed stands, SOC and most nutrients and their bioavailability were higher in rhizosphere soils, reflecting the positive effects of root activity in mixed plantations.

**Table 3:** Physicochemical properties and nutrient content of soil in different planting patterns.

Planting patterns	PP			MPE			MPD		
Soil collection locations	Rhizosphere soil	Bulk soil (0–20 cm)	Bulk soil (20–40 cm)	Rhizospher e soil	Bulk soil (0–20 cm)	Bulk soil (20–40 cm)	Rhizosphere soil	Bulk soil (0–20 cm)	Bulk soil (20–40 cm)
Electrical conductivity, EC (μs·cm^−1^)	38.70 cd	48.93 b	42.64 bc	64.67 a	33.33 de	37.58 cd	37.15 cd	28.07 ef	22.00 f
Water content, WC (%)	9.47 f	13.02 cd	13.19 cd	15.07 bc	17.11 ab	17.53 a	10.09 ef	12.90 d	11.80 de
pH	3.49 ab	3.27 b	3.60 a	3.61 a	3.54 a	3.47 ab	3.47 ab	3.54 a	3.60 a
Soil organic carbon, SOC (g·kg^−1^)	16.06 e	5.41 g	4.32 g	33.21 a	18.77 d	11.24 f	27.71 c	17.86 d	31.70 b
Total nitrogen, TN (g·kg^−1^)	2.43 abc	1.50 cd	1.04 d	3.17 a	2.69 ab	1.66 cd	3.27 a	2.05 bc	2.62 ab
Total phosphorus, TP (g·kg^−1^)	0.89 a	0.53 b	0.47 b	0.32 c	0.32 c	0.22 c	0.53 b	0.34 c	0.32 c
Total potassium, TK (g·kg^−1^)	6.70 abc	7.48 abc	3.69 bc	6.58 abc	9.31 ab	10.31 a	6.54 abc	12.59 a	11.66 c
Nitrate nitrogen, NN (mg·kg^−1^)	13.16 c	8.51 e	7.62 e	13.58 bc	11.28 cd	9.96 de	20.44 a	15.76 b	13.07 c
Ammonium nitrogen, AN (mg·kg^−1^)	6.43 bc	7.66 b	11.16 a	5.40 cd	4.37 de	3.60 de	4.74 cde	3.30 e	3.72 de
Available nitrogen, ANN (mg·kg^−1^) (Equation 1))	19.59 b	16.17 bcd	18.77 bc	18.98 bc	15.65 cd	13.55 d	25.18 a	19.05 bc	16.79 bcd
Available phosphorus, AP (mg·kg^−1^)	5.10 b	1.06 d	0.66 d	5.15 b	4.30 bc	3.40 c	7.38 a	4.41 bc	1.26 d
Available potassium, AK (mg·kg^−1^)	75.76 c	37.34 e	37.44 e	75.76 c	37.68 e	35.11 e	108.78 b	64.15 d	35.43 a
Exchangeable iron, Fe (mg·kg^−1^)	148.95 e	48.50 g	31.12 g	300.12 b	335.06 a	265.53 c	189.37 d	115.28 f	197.05 d
Exchangeable aluminum, Al (mg·kg^−1^)	798.77 c	729.00 c	708.50 c	1,244.05 ab	667.59 cd	359.74 d	663.51 cd	1,451.40 a	917.18 bc
Bioavailability of nitrogen, BN ((Equation 5)	0.82 b	1.11 b	1.92 a	0.60 b	0.58 b	0.82 b	0.77 b	0.93 b	0.67 b
Bioavailability of phosphorus, BP (Equation 6)	0.58 d	0.21 e	0.14 e	1.60 ab	1.35 bc	1.80 a	1.40 bc	1.28 c	0.40 de
Bioavailability of potassium, BK (Equation 7)	1.17 bc	0.53 c	1.35 bc	1.15 bc	0.42 c	0.39 c	1.69 b	0.51 c	3.00 a

Different lowercase letters indicate significant differences (*p* < 0.05) between soil types in the three planting patterns. Data are means (SD) (*n* = 3). PP, pure Parashorea chinensis (Pc) plantations; MPE, Pc trees in the mixed plantations of Pc and *Eucalyptus grandis × E. urophylla*; MPD, Pc trees in the mixed plantations of Pc and Dalbergia odorifera. The results of the three-way ANOVA showed that the three variable factors (planting pattern, soil type, and soil layer) had a significant effect on the majority of the environmental indicators, with a main effect, and their two-by-two interaction was also significant (*p* < 0.01), the results of which are presented in Supplementary Table S1.

### 3.2 Soil enzyme activity, microbial biomass and phosphorus fractions

**Figure 2:**
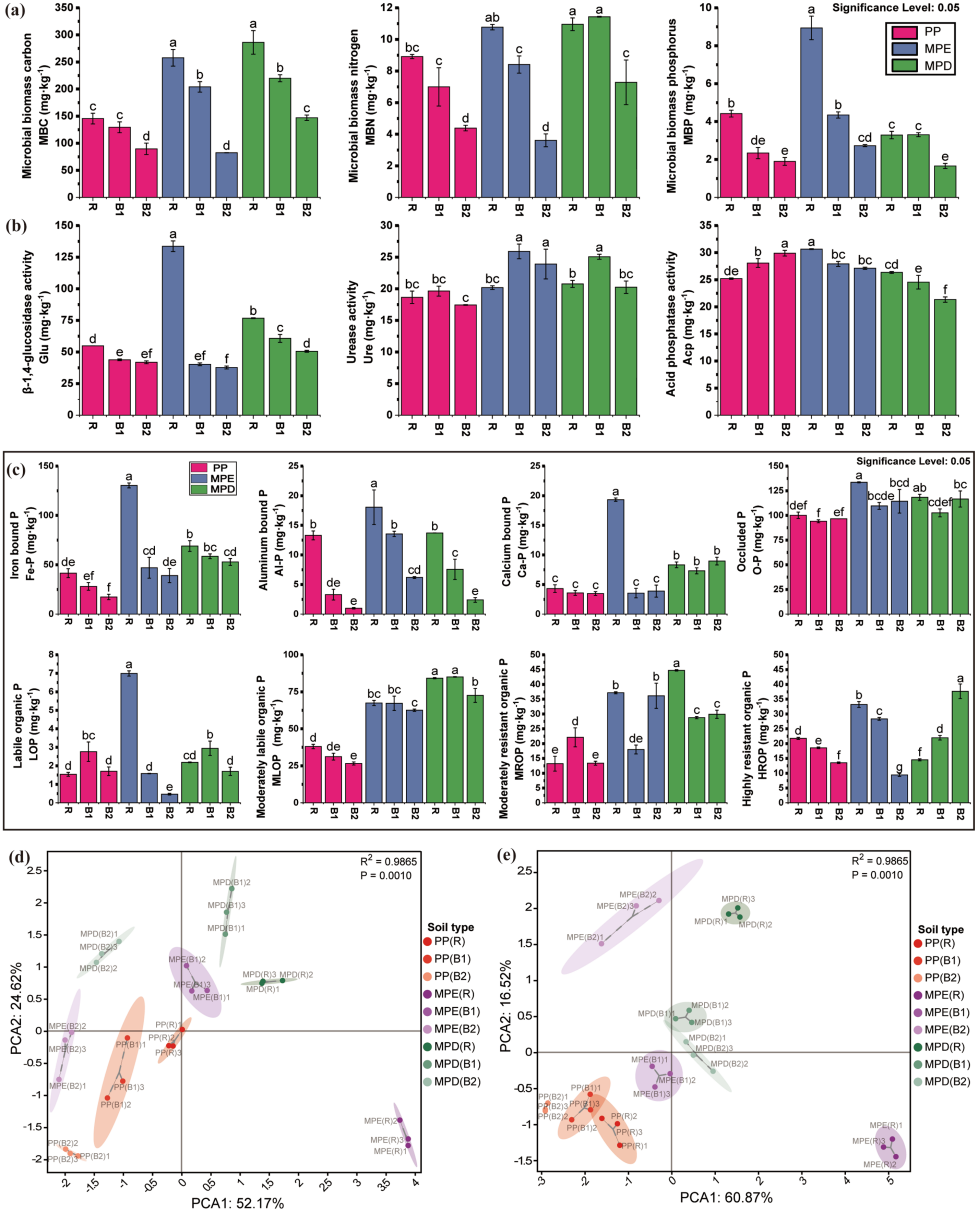
Soil microbial biomass (a), enzyme activities (b) and its principal component analysis (d), phosphorus fractions (c) and its principal component analysis (e). The meanings expressed by PP, MPE, MPD, R, B1, and B2 are the same as in the caption of Figure 1. (d): the 3 environmental variables with the largest absolute values of loadings for PC1 were β-1,4-glucosidase activity, MBC and MBP, and for PC2 were acid phosphatase activity, MBP and β-1,4-glucosidase activity; (e): the three environmental variables with the largest absolute values of loadings for PC1 were HROP, MLOP and MROP, and for PC2 were HROP, LOP and Ca-P.

Microbial biomass C, N, and P (MBC, MBN, and MBP) were higher in mixed than in pure stands in most cases (Figure 2a), and the mixed stands increased the activities of β-1,4-glucosidase, urease, and acid phosphatase in rhizosphere soils (Figure 2b). It was shown that the mixed planting patterns promoted microbial population richness and enzyme activities and optimized soil biogeochemical processes. The MBC, MBN, MBP and P fractions (either inorganic P or some fractions of organic P) showed a rhizosphere aggregation effect with the pattern of rhizosphere soil > bulk soil (0–20 cm) > bulk soil (20–40 cm). This proves that the rhizosphere microenvironment has a strong influence on nutrient accumulation and microbial activity.

Inorganic P fractions were higher in mixed stands than in pure stands, especially significantly highest in rhizosphere of MPE stands, suggesting that mixed plantations may have promoted the transformation and storage of inorganic P. The inorganic P content in the combined state with Fe, Al and Ca were all higher in rhizosphere soils than in bulk soils, and higher in surface soils than in subsoils, suggesting that the rhizosphere environment is favorable for the accumulation of inorganic P. Organic P fractions varied drastically between patterns, with the moderately labile organic P (MLOP), moderately resistant organic P (MROP) and highly resistant organic P (HROP) significantly higher in mixed stands than in pure stands in most cases, suggesting that mixed planting increased the content of these fractions, especially in MPD stands. Changes in organic P fractions were more complex and were influenced by both mixed planting patterns and tree species composition, suggesting that mixed plantations have the potential to enhance soil organic P content (Figure 2c). Principal component analysis further confirmed that microbial biomass, enzyme activities and P fractions were significantly different between mixed and pure plantations (*R*^2^ = 0.9865, *p* = 0.001). In particular, the rhizosphere region showed more active P conversion activity, further emphasizing the central role of the rhizosphere in soil nutrient cycling (Figure 2d).

### 3.3 Structural composition of soil microbial communities

**Figure 3:**
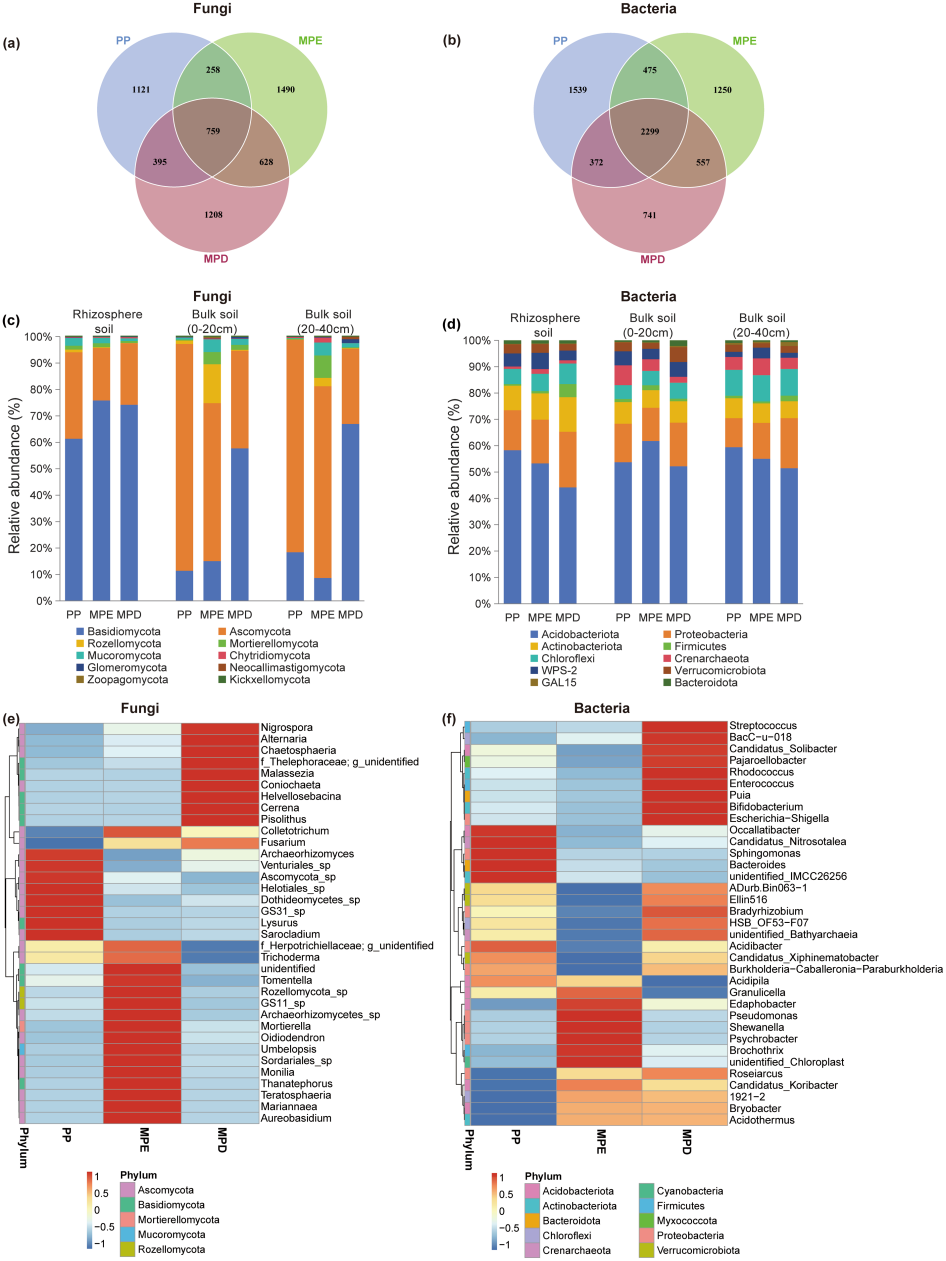
Taxonomic composition of soil fungi and bacteria under different planting patterns. The meanings expressed by PP, MPE, and MPD are the same as in the caption of Figure 1. OTU Venn diagrams (a,b), histograms of relative abundance of dominant clades (c,d), cluster analyses of the top 35 genera (e,f).

In the rhizosphere of *Parashorea chinensis*, the α-diversity indices of fungi and bacteria were higher in mixed stands than in pure stands (Table 4). The OTU numbers of fungal was higher in the two mixed stands than in the pure stands, but the OTU numbers of bacterial was higher in the pure stands (Figures 3a,b). This suggests that the fungal community is more sensitive to changes in tree species diversity. Interestingly, the OTU numbers of fungal shared by the three planting patterns (759) was smaller than any of them, but the opposite was true for bacteria. This suggests that fungal communities are more specialized across planting patterns, whereas bacterial communities have a wider range of shared core members. Fungal communities of Basidiomycota were dominant in rhizosphere soils, whereas Ascomycota was more common in bulk soils, which may be related to the preference of different fungal taxa for the rhizosphere microenvironment. Bacteria did not differ significantly between rhizosphere and non-rhizosphere, with Acidobacteriota consistently dominating, followed by Proteobacteria (Figures 3c,d). Cluster analyses further showed that both fungal and bacterial species composition in mixed plantations showed higher complexity at the phylum and genus levels, and that the species composition was more complex in mixed stands (Figures 3e,f).

**Table 4:** The α diversity index of bacteria and fungi.

Microbes		Fungi		Bacteria	
Alpha diversity index		Chao1	Ace	Chao1	Ace
Rhizosphere soil	PP	586.09 ± 223.35	595.37 ± 237.96	1,653.42 ± 247.29	1,676.11 ± 228.93
	MPE	682.31 ± 143.93	695.71 ± 162.41	1,816.49 ± 305.41	1,897.01 ± 288.01
	MPD	647.57 ± 268.08	673.09 ± 280.03	1,590.84 ± 537.03	1,636.21 ± 559.28
Bulk soil 0–20 cm	PP	783.21 ± 138.04	793.72 ± 145.97	1,672.63 ± 179.93	1,732.36 ± 203.38
	MPE	959.24 ± 833.56	812.16 ± 558.36	1,881.25 ± 458.47	1,931.46 ± 466.8
	MPD	822.39 ± 284.17	824.54 ± 290.91	1,729.64 ± 391.58	1,782.81 ± 404.06
Bulk soil 20–40 cm	PP	648.91 ± 145.46	655.03 ± 145.85	1,884.85 ± 541.45	1,940.07 ± 546.23
	MPE	1,229.33 ± 337.47	1,249.27 ± 330.71	1,894.61 ± 488.55	1,952.79 ± 492.85
	MPD	700.8 ± 132.03	708.97 ± 135.66	1,663.87 ± 169.77	1,703.15 ± 142.26

Data in the table are Mean ± SD.

### 3.4 Soil microbial community diversity

**Figure 4:**
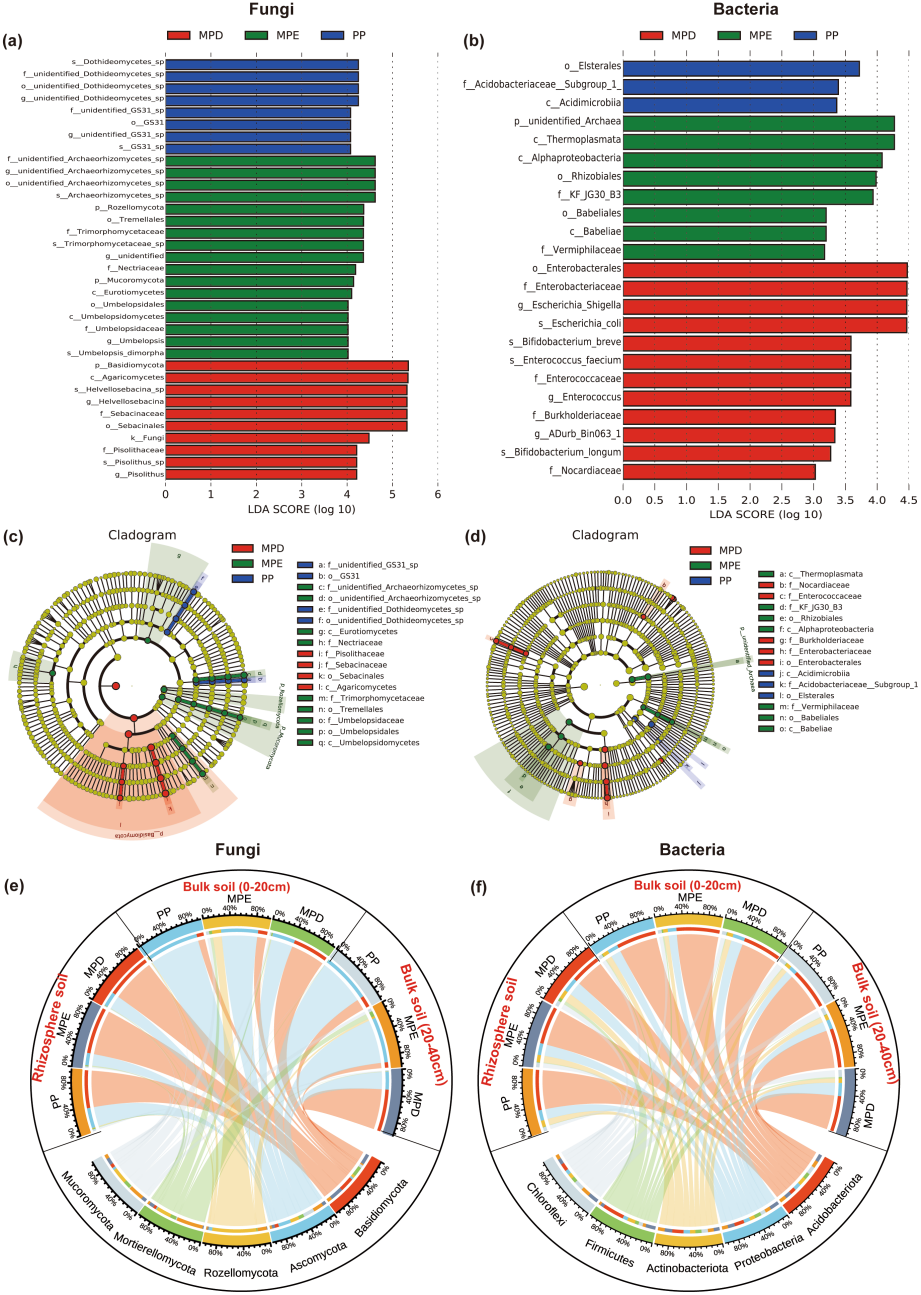
LDA Effect Size analysis and sample distribution of fungal and bacterial communities. Histograms of the distribution of LDA values for fungi and bacteria **(a, b)**, cladograms of LEfSe **(c, d)** and sankey diagrams of the distribution of dominant microbial phyla on different planting patterns **(e, f)**. Note: The meanings expressed by PP, MPE, and MPD are the same as in the caption of Figure 1.

Species with significant differences in abundance between planting patterns were detected by LEfSe (LDA Effect Size) and the effect size (LDA Score) of differentially significant species was assessed. The results showed that significant species of fungi and bacteria were not only more numerous in mixed plantations than in pure plantations, but also had a higher proportion of LDA Score > 4 (Figures 4a,b), and the evolutionary branching diagrams further demonstrated that there were more differentially significant species in mixed stands (Figures 4c,d). The high predominance of the dominant fungus Basidiomycota in rhizosphere soils reflects its important role in nutrient cycling, whereas the dominance of Ascomycota in bulk soils may be related to different ecological niche preferences. Rozellomycota and Mortierellomycota were more likely to appear in MPE. Among the dominant bacterial communities, Acidobacteriota and Proteobacteria were dominant and evenly distributed in both rhizosphere soil and bulk soil, pure and mixed plantation, suggesting that their ecological niches overlap (Figures 4e,f).

**Figure 5:**
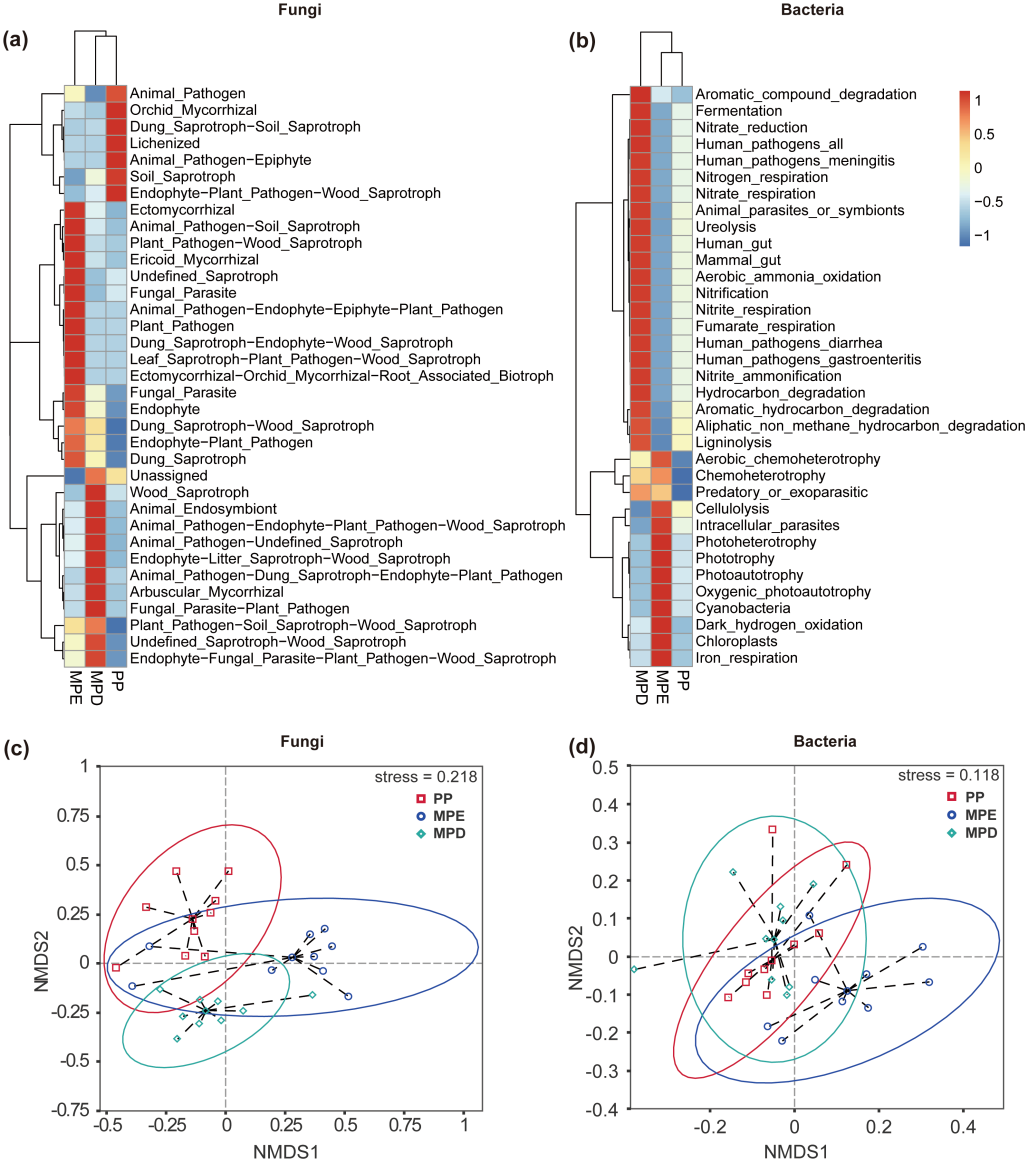
Ecofunctional clustering **(a, b)** and NMDS analysis **(c, d)** of fungal and bacterial communities. Note: The meanings expressed by PP, MPE, and MPD are the same as in the caption of Figure 1. Ellipses indicate 95% confidence intervals.

The results of cluster analysis showed that the ecological function richness of fungal and bacterial communities was higher in mixed stands than in pure stands, and different stand types had significant effects on the ecological functions of specific microbial taxa. Specifically, fungi showed more diverse ecological functions in mixed plantations (especially MPE stands), such as Ectomycorrhizal, Ericoid Mycorrhizal, Arbuscular Mycorrhizal, and Endophyte, which are essential for plantation growth and soil health (Figure 5a). The major ecological functions exhibited by bacteria in mixed plantations were Nitrification, Ureolysis and Respiration (Figure 5b). These processes are essential for soil nutrient cycling and energy flow. Non-metric multidimensional scaling (NMDS) analysis further demonstrated that there were significant differences in the diversity of soil microbial communities in the rhizosphere in pure and mixed stands (Figures 5c,d).

### 3.5 Network analysis of key microbial communities

**Figure 6:**
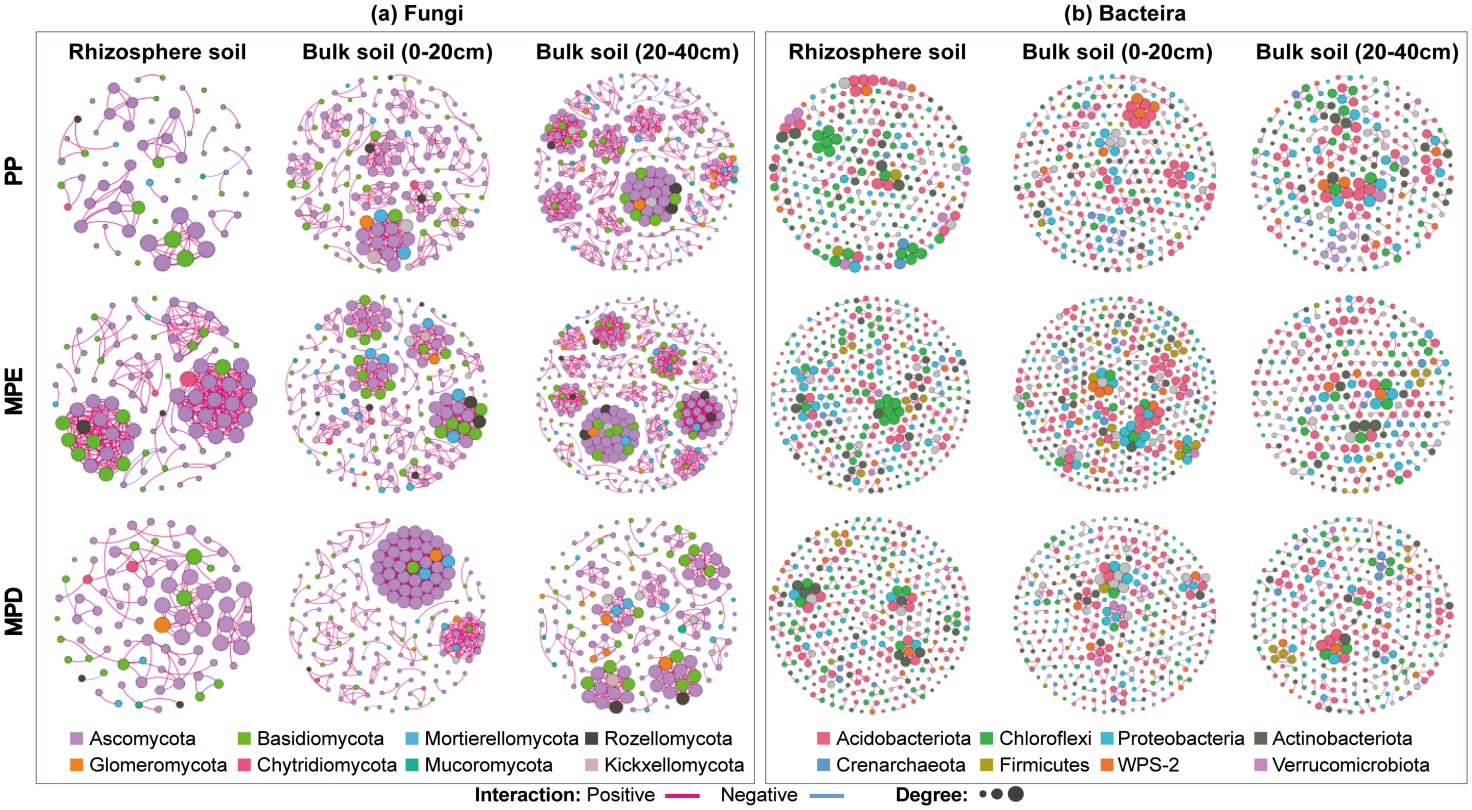
Co-occurrence networks of key soil fungal **(a)** and bacterial **(b)** communities under different planting patterns. Note: The size of each node is proportional to the number of connections. Each node is marked at the phylum level. Every two nodes are connected by edges, and the two colors of the edges represent positive or negative correlation. PP, MPE and MPD are the same as in the caption of Figure 1.

The results of co-occurrence network analysis showed that the complexity of the microbial community network, characterized by Average degree (AD), produced a large divergence between planting patterns, with differences in the spatial distribution of microbial core species and microbial network module structure in the soil (Figure 6). Interaction relationships among key species in the fungal network and their co-occurrence complexity were significantly higher in mixed stands, especially MPE stands, than in pure stands. It indicates that mixed planting can increase the number of key species that are important for network structure and function. In addition, the abundance of fungi and bacteria as well as interaction or co-occurrence relationships were lower in rhizosphere soils than in bulk soils as shown by the performance of Node and Edge (Table 5).

**Table 5:** Topological properties of co-occurrence networks.

Microbes	Planting patterns	Soil collection locations	Nodes	Edges			Average degree	Modularity	Density
				Total	Positive (%)	Negative (%)			
Fungi	PP	Rhizosphere soil	66	70	98.57	1.43	2.121	0.852	0.033
		Bulk soil (0–20 cm)	163	371	100.00	0.00	4.552	0.881	0.028
		Bulk soil (20–40 cm)	254	964	99.59	0.41	7.591	0.834	0.030
	MPE	Rhizosphere soil	96	393	98.98	1.02	8.188	0.681	0.086
		Bulk soil (0–20 cm)	188	496	100.00	0.00	5.277	0.875	0.028
		Bulk soil (20–40 cm)	301	1,508	99.73	0.27	10.020	0.795	0.033
	MPD	Rhizosphere soil	83	92	97.83	2.17	2.217	0.904	0.027
		Bulk soil (0–20 cm)	173	985	99.70	0.30	11.387	0.364	0.066
		Bulk soil (20–40 cm)	167	335	99.40	0.60	4.012	0.883	0.024
Bacteria	PP	Rhizosphere soil	282	242	64.88	35.12	2.378	0.977	0.006
		Bulk soil (0–20 cm)	348	351	72.36	27.64	2.017	0.974	0.006
		Bulk soil (20–40 cm)	342	333	71.77	28.23	1.947	0.965	0.006
	MPE	Rhizosphere soil	311	327	78.29	21.71	2.103	0.967	0.007
		Bulk soil (0–20 cm)	383	448	87.50	12.50	2.339	0.973	0.006
		Bulk soil (20–40 cm)	333	369	87.26	12.74	2.216	0.953	0.007
	MPD	Rhizosphere soil	257	250	84.80	15.20	1.946	0.967	0.008
		Bulk soil (0–20 cm)	281	219	71.23	28.77	1.559	0.976	0.006
		Bulk soil (20–40 cm)	303	288	77.78	22.22	1.901	0.965	0.006

Average clustering coefficient: Local connectivity of microbial connections. Average path length: Average distance between microbial connections. A smaller value means all the nodes in the network are closer. Modularity: Degree of nodes tending to differentiate into different network modules. Density: It represents the level of interconnectedness among microbial species. It is calculated as the ratio of the actual number of connections to the maximum possible connections in the network. The meanings expressed by PP, MPE, and MPD are the same as in the caption of Table 1.

### 3.6 Relationship between soil nutrients and microorganisms

**Figure 7:**
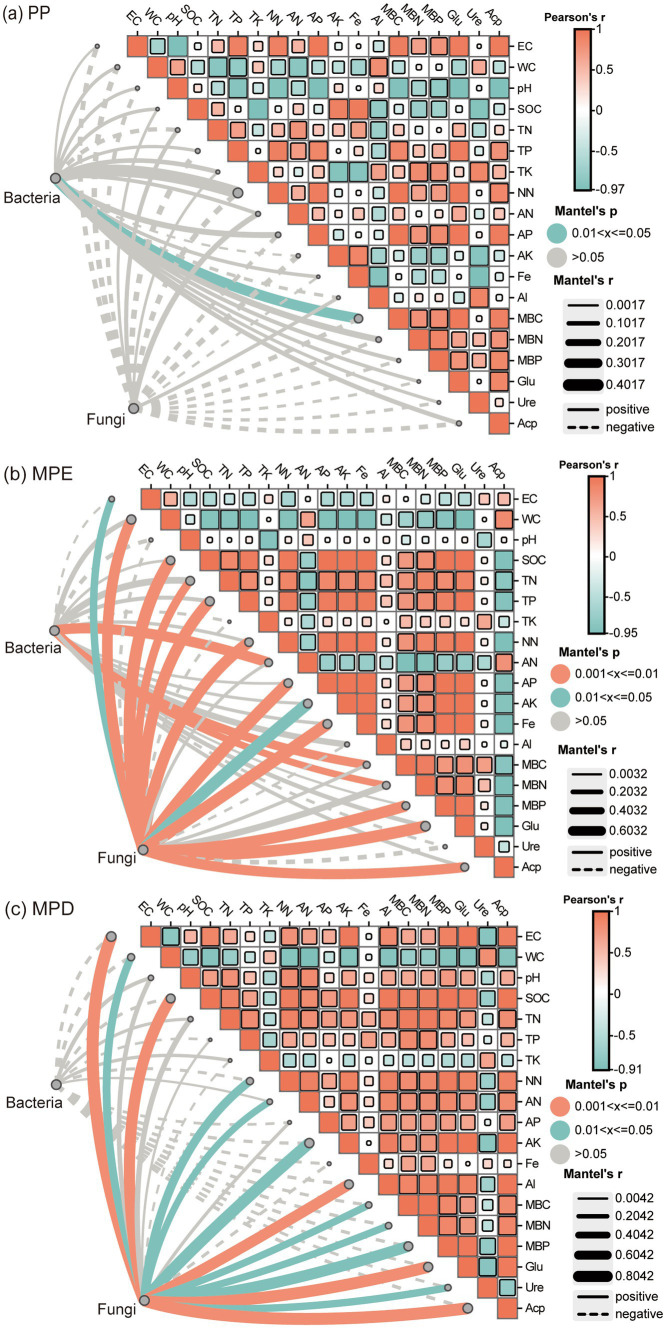
Mantel test for correlation between soil microorganisms and environmental factors. Note: **(a)**, Planting pattern PP; **(b)**, Planting pattern MPE; **(c)**, Planting pattern MPD. The meanings expressed by PP, MPE, and MPD are the same as in the caption of Figure 1. EC, electrical conductivity; WC, water content; SOC, soil organic carbon; TN, total nitrogen; TP, total P; TK, total potassium; NN, nitrate nitrogen; AN, ammonium nitrogen; AP, available P; AK, available potassium; Glu, β-1,4-glucosidase activity; Ure, urease activity; Acp, acid phosphatase activity; PA, P availability; Al, exchangable aluminium; Fe, exchangable iron; MBC, microbial biomass carbon; MBN, microbial biomass nitrogen; MBP, microbial biomass P. Fe-P, iron-bound P; Al-P, aluminum-bound P; Ca-P, calcium-bound P; O-P, insoluble P; LOP, labile organic P; MLOP, moderately labile organic P; MROP, moderately resistant organic P; HROP, highly resistant organic P.

Mantel test results showed that in pure stands, only bacterial diversity showed a significant positive correlation with microbial biomass C (*p* < 0.05), whereas fungi were not significantly associated with all environmental factors (Figure 7a). In mixed stands, the interaction between microorganisms and environmental factors is more significant and complex. In the MPE stands, bacterial communities were highly significantly positively correlated (*p* < 0.01) with ammonium N, microbial biomass C and N, and fungal communities were highly significantly positively correlated with multiple nutrient (Figure 7b). Within the MPD stand, while none of the bacterial correlations with environmental factors were significant, fungi were highly significantly correlated with soil conductivity, organic C, exchanged Al, β-1,4-glucosidase and acid phosphatase (Figure 7c). It indicated that soil microorganisms in mixed forests were more vigorous and sensitive to environmental responses.

**Figure 8:**
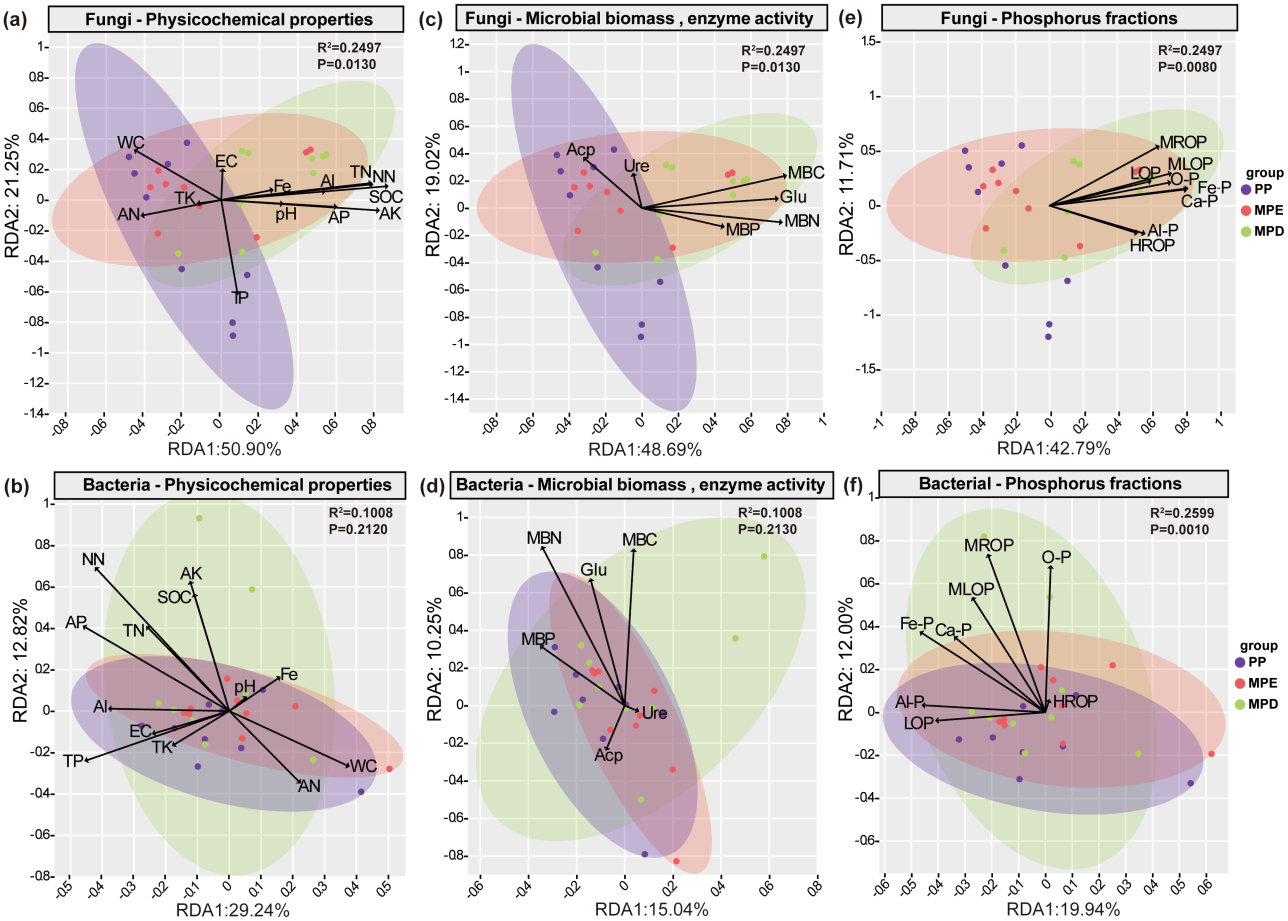
Redundancy analysis of soil microorganisms with environmental factors. RDA of fungi **(a)** and bacteria **(b)** with physicochemical properties, fungi **(c)** and bacteria **(d)** with microbial biomass and enzyme activities, and fungi **(e)** and bacteria **(f)** with phosphorus fractions, respectively. Note: The abbreviations in the figures convey the same meaning as in the caption of Figure 7.

Redundancy analysis further showed that the effects of soil physicochemical properties, microbial biomass and enzyme activities, and P fractions on the structural variability of the fungal community reached significant levels (*p* < 0.05), however, only the P fractions affected the bacterial community at highly significant levels (*p* < 0.01). The total N, ammonium N, nitrate N, organic C, available K, microbial biomass C and acid phosphatase activity were the key factors driving changes in fungal communities; The total P, available P, microbial biomass N and P and acid phosphatase activity were the key physico-chemical and biological factors driving changes in bacterial communities (Figures 8a–d). Phosphorus in the Fe, Al, and Ca-bound states, as well as moderately labile organic phosphorus, were the key P factors driving changes in fungal and bacterial communities (Figures 8e,f).

## 4 Discussion

### 4.1 Effects of mixed planting on soil environmental factors in Parashorea chinensis plantations

Red soils in the tropics and subtropics are inherently acidic (pH < 6.0), and nutrient limitation occurs when soil acidification is severe enough to affect the bioavailability of nutrients (Lu et al., 2014). The soil pH of all sample plots in this study was below 4.0, which implies that the study area is located in a region of strongly acidic soils, where P and K are more easily lost or immobilized. However, we found that mixed forests show potential to alleviate soil nutrient limitation by increasing soil fertility through increased organic C and N content, total and effective state content of N and K, and bioavailability of P. This may be due to the fact that mixed plantations promote the return of nutrients from tree litter and the accumulation of soil organic matter (Deng et al., 2016; Kooch and Bayranvand, 2017; Xu et al., 2021). In contrast to the performance of pure forests, rhizosphere soils in mixed forests had significantly lower water content but higher electrical conductivity. This reflects the more complex root structure and higher water use efficiency of mixed plantations. Moreover, the significant increase in electrical conductivity in rhizosphere soils of mixed stands may be attributed to the combined effects of chemosensitivity, root secretions, and microbial activities among the mixed species (Nsengimana et al., 2024). Specifically, the wide root distribution and high litterfall of *Eucalyptus grandis × E. urophylla* and *Dalbergia odorifera* in the mixed stands had a greater influence on the spatial distribution and the rhizosphere effect of soil nutrients (Xiang et al., 2021). Moreover, *Dalbergia odorifera*, as a N-fixing woody legume, fixes atmospheric N through the symbiotic action of rhizobacteria and can transfer N to other tree species, thus enhancing N cycling and transfer (Yao et al., 2019). In addition, N fixation by legume rhizosphere can indirectly increase the bioavailability of P and K and promote their uptake by plants (Duchene et al., 2017). These findings provide useful tree species options for the construction of *Parashorea chinensis* mixed plantations. Although the above study verified our first hypothesis that mixed plantations changed the soil nutrient and spatial distribution structure and improved the soil physicochemical properties. However, the problem of strongly acidic soil still needs to be solved, and it can be improved in the future by adding lime and other measures.

The assembly process of fungal community were affected by soil enzyme activities (Ma et al., 2024). In this study, it was found that compared to pure stands, mixed planting increased soil microbial biomass C, N, P and β-1,4-glucosidase and urease activities, altered their spatial distribution patterns and optimized soil biogeochemical processes. This may be related to the more complex ecological niche and richer resources provided by the root system of mixed forests. This implies a change in the way soil C, N, and P are cycled and utilized, a change that may favor the improvement of soil nutrient effectiveness and promote the transformation and accumulation of P among its components (Duan et al., 2019). Statistically significant correlation between phosphorus fraction effectiveness and acid phosphatase activity (Giles et al., 2018; Fu et al., 2020). We found that the rhizosphere accumulation of inorganic P fractions was significantly higher in mixed forests than in pure forests, and the variation in organic P fractions was complex but with high potential for enhancement. Higher soil acid phosphatase activity in P-poor sites may accelerate the release of P into soil solutions. However, it has also been suggested that soil physicochemical processes determine P bioavailability, and that mixed forests influence soil respiration and microbial amounts of P (Bünemann et al., 2016). Other studies in the region have also shown that soil P effectiveness is mainly associated with changes in moderately active P concentrations in pure forests and highly stable P concentrations in mixed forests (Yue et al., 2022). These results above validate our hypothesis that mixed forests increase microbial biomass and key enzyme activities, enhancing P conversion and thus alleviating nutrient limitation.

This study also found that O-P and MLOP dominated P fractions, consistent with their instability and difficulty in decomposition (Gatiboni et al., 2017). Another study in the subtropical mountains of China concluded that occluded and organic P was the dominant P fraction only in a low-P site (Fu et al., 2020). Strong correlations exist between exchangeable aluminum (Al) and exchangeable iron (Fe) content and P effectiveness in acidic soils (Zarif et al., 2020). Mixed plantations exhibit differing correlations between available P, Fe^3+^, and Al^3+^ content in acidic soils, likely due to altered physicochemical properties (Deforest, 2020). Root exudates release bound P from soil surfaces and cations like Ca^2+^, Al^3+^, and Fe^3+^ without directly increasing biologically-available P, affecting P sequestration and effectiveness (Mezeli et al., 2020). These results support the positive effects of mixed forests on soil P morphological transformation and P activation in the rhizosphere of *Parashorea chinensis* plantation.

### 4.2 Effects of mixed planting on soil microorganisms in *Parashorea chinensis* plantations

Plant species and soil collection locations together determine the structure and function of rhizosphere microbial communities (Berg and Smalla, 2009). The composition of microbial communities in forest soils is mainly determined by dominant tree species, and fungal and bacterial communities respond differently to dominant tree species, and they affect the soil to different degrees (Bai et al., 2023). In this study, we found that OTU numbers of fungi were higher in mixed stands, whereas bacteria were more abundant in pure stands. This suggests that fungal communities are more sensitive to changes in tree species diversity, possibly due to differences in root secretions and microenvironments of different tree species providing more ecological niches for fungi (Figures 3a,b). In contrast, bacterial communities may be more adapted to stable environments formed by a single tree species, or there may be a wide range of adaptations among different tree species. The composition of the microbial community in forest soils is largely determined by the dominant tree species, with fungal and bacterial communities responding differently to the dominant species, and the extent to which they affect the soil varies (Urbanová et al., 2015). The results of the OTU numbers we found to be shared by different planting patterns also suggest a high degree of specialization of fungal communities in specific ecological niches. Bacterial communities, on the other hand, have a wider range of shared core members and may possess more general and flexible survival strategies. The present study demonstrated that fungal communities are more susceptible to rhizosphere environments than bacteria, and that microbial diversity and species composition complexity were higher in rhizosphere soils of mixed stands than in pure stands. Mixed planting significantly increased the relative abundance of the dominant fungus Basidiomycota and bacteria Acidobacteriota (Figures 3c–f). This may be due to the fact that eucalyptus and leguminous species *Dalbergia odorifera* in mixed stands exist in more symbiotic relationships with soil microorganisms, which can provide a wider range of ecological niches for the microorganisms (Chou et al., 2019).

The results of LEfSe analyses showed that the proportion of significantly different species (Biomaker) with LDA Score > 4 was also higher, suggesting that microbial communities in mixed forests have more significant diversity differences and greater ecological effects (Figures 4a,b). This may be related to more complex ecological niches and richer resource conditions in mixed forests, supporting the theory that biodiversity promotes ecosystem stability. The cladogram visually demonstrated significant differences in microbial communities between mixed and pure stands, especially for species with higher LDA Score, which provides an important basis for understanding the effects of different stands on microbial community structure (Figures 4c,d). The results of cluster analyses revealed the dominance of mixed forests over pure forests in terms of ecological functional richness of fungal and bacterial communities, and that different stand types had significant effects on the ecological functions of specific microbial taxa (Figures 5a,b). Notably, bacterial ecological functions in MPD stands reached the highest level of significance, suggesting that specific mixed patterns may be favorable to the enhancement of bacterial functional diversity. NMDS analyses reinforced the significant difference in functional diversity of soil microbial communities in the rhizosphere between pure and mixed stands, which not only revealed the profound effect of stand type on microbial community structure, but also emphasized the role of mixed forests potential advantages in maintaining and enhancing soil microbial functional diversity (Figures 5c,d). Co-occurrence network analysis of microorganisms showed that mixed forests possessed more nodes of the fungal Mortierellomycota network (Figure 6), suggesting the presence of more fungi involved in P catabolism and transport in mixed forest soils, since Mortierellomycota is widely recognized as an important component of the tufted mycorrhizal fungi that help plants take up P and other mineral nutrients while simultaneously obtaining carbon from the plants to obtain carbon sources, and is a major predictor of P cycling (Xu et al., 2021). Mixed plantations of *Parashorea chinensis* and eucalyptus were more favorable for maintaining complex and diverse network relationships among fungal communities than mixed plantations, with the particularly rhizosphere soils, and improved the correlation between the number of key species and microbial networks. This may be due to the diversity of tree species in mixed forests, which gain rapid growth by virtue of their deep and large root systems, which have a greater impact on the soil biotic and abiotic environments (Baumert et al., 2021). And the mixing of two species optimizes and complements the spatial structure and nutrient utilization of soil, as well as the function and structure of microbial communities, and improves the stability of the soil microbiology, especially in the active rhizosphere region. Long-term organic matter transfer by deep-rooting plants like eucalyptus, such as trees might thus strongly affect aggregation in subsoils (Blanco-Canqui and Lal, 2004). Although these findings provide important insights into understanding the functional properties of microbial communities under different stand types, studies still need to consider the interactions of additional environmental factors and what long-term monitoring data reveal about the dynamics of microbial communities.

### 4.3 Effect of mixed planting on the relationship between soil microbial communities and environmental indicators

The mixed planting significantly affects soil physicochemical properties and the structural and functional diversity of microbial communities (Zhang et al., 2021). In this study, it was found that microbial communities in mixed stands (MPE) had higher correlations with environmental factors compared to pure stands, but microbes in mixed stands (MPD) had lower correlations with environmental factors, especially in terms of fungal communities. This suggests that tree species composition has an important influence on the relationship between microbial communities. Other studies have found that when microbial changes are induced by mixed planting, fungal diversity is more sensitive to tree species than bacteria, which are more susceptible to microhabitat changes (Urbanová et al., 2015), which may be due to the fact that fungi are more affected by changes in apoplastic inputs, and this is in line with our findings. It may also be due to the fact that stands with a more complex tree species composition produce more diverse apoplastic material and a wider range of species of root secretions, while fungi are able to take up more nutrients, leading to the development of a more diverse microbial community structure and function (Klimek et al., 2016).

Significant changes in microbial communities occur with the decomposition of apoplastic leaves of various plants (Liu et al., 2022). The results of Mantel test in this study showed stronger interaction and association between microorganisms and environmental factors in mixed stands compared to pure stands (Figure 7). In particular, In this study, the compositional structure of the microbial community in the mixed forests of *Parashorea chinensis* and eucalyptus was significantly correlated (*p* < 0.01) with most of the environmental factors. The reason may be that apoplasts of mixed forests have higher fungal and bacterial abundance as well as microbial community diversity than apoplasts of pure forests, and they have significantly different microbial community compositions (Patoine et al., 2017). Interestingly, fungal communities were significantly more correlated with environmental factors compared to bacteria. Soil organic C content, β-1,4-glucosidase and acid phosphatase activity were all highly significantly and positively correlated with fungal community diversity in both mixed forests. This may be because microbial limitation of C and P is regulated by the stoichiometric ratios of the relevant cyclic enzymes (Yang et al., 2023). Biotic and abiotic factors shape the rhizosphere microbiome (Pii et al., 2016). Redundancy analyses indicated that organic C, effective N, microbial biomass C and N, β-1,4-glucosidase, and insoluble P were the key factors driving changes in the fungal and bacterial communities together (Figure 8). The biotic factor aspect could be the direct interaction between apoplastic production and C, N, and microorganisms in root deposits that are symbiotic with roots and have the ability to transform C and N (Urbanová et al., 2015). And abiotic factors may be richer biomass in the soil and the C sources in root secretions (Yu et al., 2018).

## 5 Conclusion

All the stands in this study had strongly acidic (pH < 4) soils. The mixed plantations significantly outperformed the pure plantations in terms of soil physico-chemical properties, nutrients and their availability, especially in terms of increased soil water content, organic carbon, total N, K and available nutrients. The spatial distribution of microbial biomass, enzyme activities and P fractions in mixed stands was significantly affected, with rhizosphere soils showing higher nutrient bioavailability and P conversion activity. Fungal communities were more sensitive to tree species composition and had higher diversity in the rhizosphere of mixed plantations, whereas bacterial community structure did not show strong mixing and rhizosphere effects. The species composition of fungi was more complex than that of bacteria in mixed forests, and there were more differential species present in mixed stands than in pure stands, and the diversity of soil ecological functions embodied by these species was significantly higher than in pure stands. The correlation between microbial communities and environmental factors was higher in mixed stands than in pure stands, and all soil environmental factors had a significant effect on the structure of fungal communities, but bacterial communities were only significantly affected by phosphorus fractions. In summary, the mixed planting pattern not only improves soil quality, but also enhances the diversity and activity of soil microbial communities and their connection with the soil environment. In particular, the microbial community in the rhizosphere soil plays an important role in soil nutrient availability and P transformation. Therefore, when afforestation is carried out in acidic, low-phosphorus red soils, it may be helpful to optimize nutrient effectiveness and microbial communities through a rational mix of tree species, and special attention should be paid to this in the practice of converting pure forests to mixed forests. And we recommend the establishment of mixed plantations of *Parashorea chinensis* and *Eucalyptus grandis × E. urophylla*, as well as the introduction of tree species such as *Dalbergia odorifera*, which contribute to the improvement of soil fertility and the regulation of interspecific relationships, in order to fulfill better soil ecological functions and improve the sustainable management of plantation forests in nutrient-limited areas.

## Data Availability

The original contributions presented in the study are publicly available. This data can be found here: The National Center for Biotechnology Information https://www.ncbi.nlm.nih.gov/ accession number: PRJNA1155876.

## Supplementary material

SUPPLEMENTARY FIGURE S1 Schematic map of the location of the study site and sample plots.
