# Supplementary figures and images for: Effect of mixed planting on soil nutrient availability and microbial diversity in the rhizosphere of Parashorea chinensis plantations
Source: Front Microbiol. 2024 Oct 15;15:1464271. doi: 10.3389/fmicb.2024.1464271 (PMC11520325; doi:10.3389/fmicb.2024.1464271)

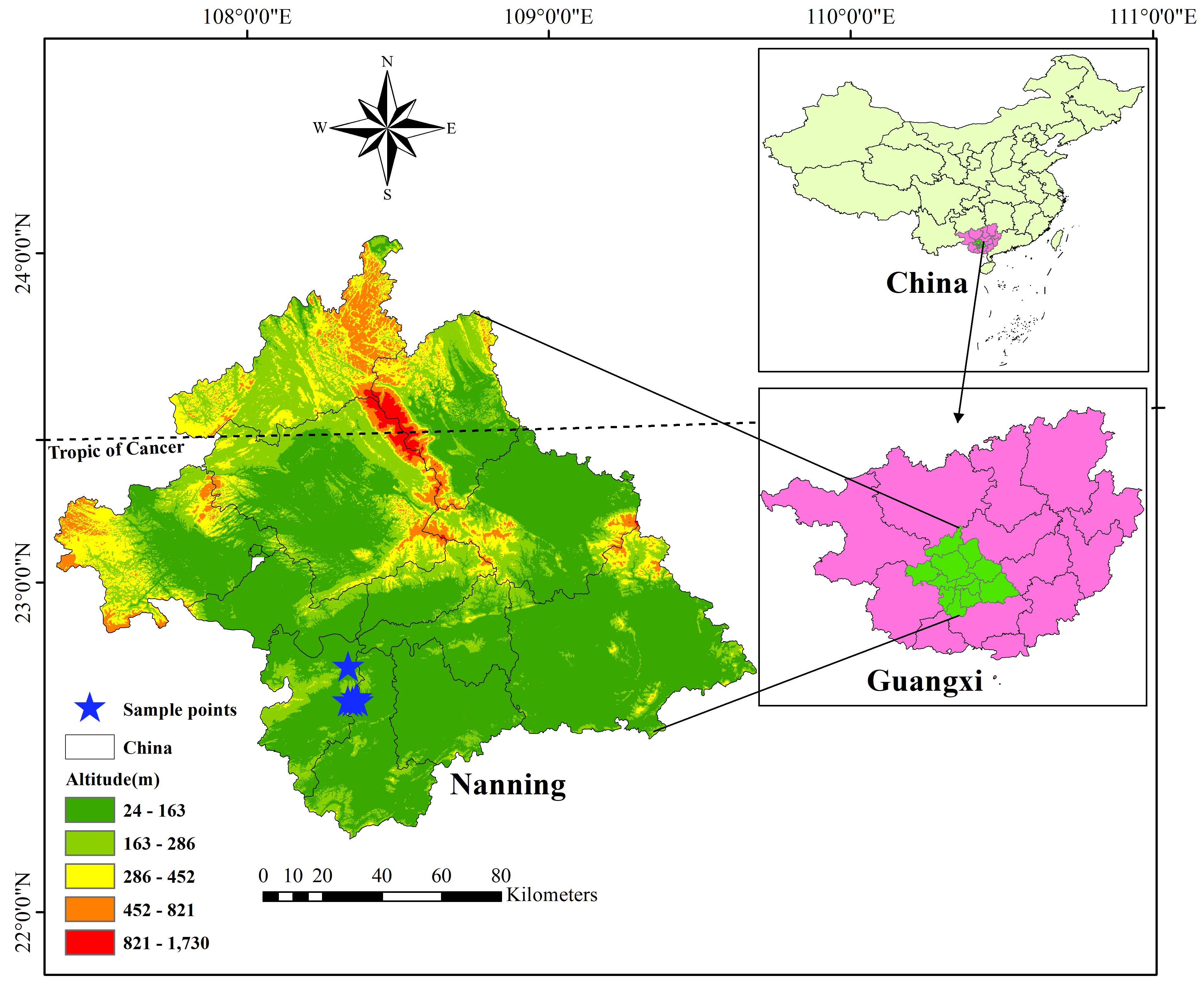

Supplement: SUPPLEMENTARY FIGURE S1 — Schematic map of the location of the study site and sample plots. [file Image_1.jpeg]
