# Supplementary material for: Effect of mixed planting on soil nutrient availability and microbial diversity in the rhizosphere of Parashorea chinensis plantations
Source: Front Microbiol. 2024 Oct 15;15:1464271. doi: 10.3389/fmicb.2024.1464271 (PMC11520325; doi:10.3389/fmicb.2024.1464271)
Supplement: Supplementary file 2 [file Data_Sheet_1.pdf]

Supplementary material

Table S1 Three-way ANOVA for environmental indicators

Index	Items	Square sum	Degree of freedom	Mean square	F	P
EC	Intercept	41625.953	1	41625.953	5471.336	0.000***
	Planting pattern	1482.955	2	741.477	97.46	0.000***
	Soil type	589.551	1	589.551	77.491	0.000***
	Soil layer	3928.769	2	1964.384	258.2	0.000***
	Planting pattern * Soil type	1348.931	2	674.466	88.652	0.000***
	Planting pattern * Soil layer	1482.129	4	370.532	48.703	0.000***
	Soil type * Soil layer	38796.576	2	19398.288	2549.721	0.000***
WC	Intercept	4596.631	1	4596.631	8065.35	0.000***
	Planting pattern	137.221	2	68.611	120.386	0.000***
	Soil type	552.037	1	552.037	968.616	0.000***
	Soil layer	151.444	2	75.722	132.864	0.000***
	Planting pattern * Soil type	1.322	2	0.661	1.16	0.336
	Planting pattern * Soil layer	19.202	4	4.801	8.423	0.001***
	Soil type * Soil layer	3632.766	2	1816.383	3187.066	0.000***
pH	Intercept	321.716	1	321.716	39946.582	0.000***
	Planting pattern	0.029	2	0.014	1.798	0.194
	Soil type	19.347	1	19.347	2402.32	0.000***
	Soil layer	11.942	2	5.971	741.398	0.000***
	Planting pattern * Soil type	0.061	2	0.03	3.768	0.043**
	Planting pattern * Soil layer	0.164	4	0.041	5.09	0.006***
	Soil type * Soil layer	274.394	2	137.197	17035.403	0.000***
SOC	Intercept	9269.499	1	9269.499	33850.41	0.000***
	Planting pattern	1304.691	2	652.345	2382.239	0.000***
	Soil type	4.807	1	4.807	17.553	0.001***
	Soil layer	1440.323	2	720.162	2629.89	0.000***
	Planting pattern * Soil type	109.44	2	54.72	199.827	0.000***
	Planting pattern * Soil layer	588.96	4	147.24	537.692	0.000***
	Soil type * Soil layer	9622.774	2	4811.387	17570.252	0.000***
TN	Intercept	142.312	1	142.312	1340.339	0.000***
	Planting pattern	4.77	2	2.385	22.463	0.000***
	Soil type	0.347	1	0.347	3.268	0.087*
	Soil layer	20.388	2	10.194	96.012	0.000***
	Planting pattern * Soil type	0.316	2	0.158	1.49	0.252
	Planting pattern * Soil layer	2.137	4	0.534	5.032	0.007***
	Soil type * Soil layer	139.081	2	69.541	654.956	0.000***
TP	Intercept	5.309	1	5.309	2628.073	0.000***
	Planting pattern	0.641	2	0.32	158.575	0.000***
	Soil type	0.007	1	0.007	3.228	0.089*
	Soil layer	0.809	2	0.404	200.186	0.000***

	Planting pattern * Soil type	0.055	2	0.027	13.576	0.000***
	Planting pattern * Soil layer	0.283	4	0.071	35.079	0.000***
	Soil type * Soil layer	5.239	2	2.62	1296.782	0.000***
TK	Intercept	1455.249	1	1455.249	315.885	0.000***
	Planting pattern	27.9	2	13.95	3.028	0.074*
	Soil type	163.513	1	163.513	35.493	0.000***
	Soil layer	257.804	2	128.902	27.98	0.000***
	Planting pattern * Soil type	18.3	2	9.15	1.986	0.166
	Planting pattern * Soil layer	114.692	4	28.673	6.224	0.003***
	Soil type * Soil layer	1244.333	2	622.166	135.051	0.000***
NN	Intercept	4353.999	1	4353.999	6178.411	0.000***
	Planting pattern	207.326	2	103.663	147.1	0.000***
	Soil type	31.57	1	31.57	44.798	0.000***
	Soil layer	531.991	2	265.996	377.453	0.000***
	Planting pattern * Soil type	6.99	2	3.495	4.96	0.019**
	Planting pattern * Soil layer	40.159	4	10.04	14.246	0.000***
	Soil type * Soil layer	4152.321	2	2076.161	2946.113	0.000***
AN	Intercept	799.575	1	799.575	1588.587	0.000***
	Planting pattern	84.934	2	42.467	84.373	0.000***
	Soil type	50.195	1	50.195	99.728	0.000***
	Soil layer	14.732	2	7.366	14.635	0.000***
	Planting pattern * Soil type	23.415	2	11.708	23.26	0.000***
	Planting pattern * Soil layer	18.686	4	4.671	9.281	0.000***
	Soil type * Soil layer	685.776	2	342.888	681.246	0.000***
ANN	Intercept	8885.249	1	8885.249	5523.815	0.000***
	Planting pattern	89.994	2	44.997	27.974	0.000***
	Soil type	161.38	1	161.38	100.327	0.000***
	Soil layer	722.174	2	361.087	224.482	0.000***
	Planting pattern * Soil type	8.06	2	4.03	2.505	0.11
	Planting pattern * Soil layer	67.479	4	16.87	10.488	0.000***
	Soil type * Soil layer	8195.989	2	4097.994	2547.657	0.000***
AP	Intercept	391.719	1	391.719	1658.987	0.000***
	Planting pattern	19.477	2	9.739	41.244	0.000***
	Soil type	7.633	1	7.633	32.328	0.000***
	Soil layer	140.186	2	70.093	296.854	0.000***
	Planting pattern * Soil type	12.605	2	6.302	26.691	0.000***
	Planting pattern * Soil layer	16.46	4	4.115	17.428	0.000***
	Soil type * Soil layer	434.565	2	217.282	920.224	0.000***
AK	Intercept	117932.652	1	117932.652	16620.712	0.000***
	Planting pattern	15403.929	2	7701.965	1085.468	0.000***
	Soil type	108.889	1	108.889	15.346	0.001***
	Soil layer	11471.502	2	5735.751	808.362	0.000***
	Planting pattern * Soil type	685.354	2	342.677	48.295	0.000***

	Planting pattern * Soil layer	5457.811	4	1364.453	192.298	0.000***
	Soil type * Soil layer	121224.394	2	60612.197	8542.315	0.000***
Fe	Intercept	879706.558	1	879706.558	22260.768	0.000***
	Planting pattern	187558.408	2	93779.204	2373.061	0.000***
	Soil type	14695.174	1	14695.174	371.858	0.000***
	Soil layer	70482.944	2	35241.472	891.777	0.000***
	Planting pattern * Soil type	32991.354	2	16495.677	417.419	0.000***
	Planting pattern * Soil layer	19720.723	4	4930.181	124.757	0.000***
	Soil type * Soil layer	815224.163	2	407612.082	10314.528	0.000***
Al	Intercept	19352342.87	1	19352342.87	1311.375	0.000***
	Planting pattern	135338.33	2	67669.165	4.585	0.025**
	Soil type	878319.577	1	878319.577	59.518	0.000***
	Soil layer	2247125.096	2	1123562.548	76.136	0.000***
	Planting pattern * Soil type	1729322.913	2	864661.457	58.592	0.000***
	Planting pattern * Soil layer	1331600.874	4	332900.218	22.558	0.000***
	Soil type * Soil layer	17086949.21	2	8543474.607	578.932	0.000***
BN	Intercept	20.57	1	20.57	331.138	0.000***
	Planting pattern	1.531	2	0.765	12.321	0.000***
	Soil type	2.983	1	2.983	48.022	0.000***
	Soil layer	0.112	2	0.056	0.9	0.424
	Planting pattern * Soil type	0.352	2	0.176	2.832	0.085*
	Planting pattern * Soil layer	0.998	4	0.25	4.018	0.017**
	Soil type * Soil layer	16.095	2	8.048	129.549	0.000***
BP	Intercept	26.109	1	26.109	2457.317	0.000***
	Planting pattern	6.64	2	3.32	312.485	0.000***
	Soil type	0.271	1	0.271	25.505	0.000***
	Soil layer	3.176	2	1.588	149.466	0.000***
	Planting pattern * Soil type	0.311	2	0.155	14.622	0.000***
	Planting pattern * Soil layer	1.709	4	0.427	40.209	0.000***
	Soil type * Soil layer	24.622	2	12.311	1158.666	0.000***
BK	Intercept	69.103	1	69.103	465.467	0.000***
	Planting pattern	48.941	2	24.471	164.829	0.000***
	Soil type	10.129	1	10.129	68.229	0.000***
	Soil layer	27.445	2	13.722	92.431	0.000***
	Planting pattern * Soil type	5.547	2	2.774	18.682	0.000***
	Planting pattern * Soil layer	79.956	4	19.989	134.642	0.000***
	Soil type * Soil layer	96.845	2	48.423	326.166	0.000***
MBC	Intercept	867092.245	1	867092.245	6577.027	0.000***
	Planting pattern	45761.99	2	22880.995	173.556	0.000***
	Soil type	2409.83	1	2409.83	18.279	0.000***
	Soil layer	186160.353	2	93080.176	706.027	0.000***
	Planting pattern * Soil type	1251.823	2	625.912	4.748	0.022**
	Planting pattern * Soil layer	22777.051	4	5694.263	43.192	0.000***

	Soil type * Soil layer	856117.684	2	428058.842	3246.892	0.000***
MBN	Intercept	1873.826	1	1873.826	4031.969	0.000***
	Planting pattern	36.526	2	18.263	39.297	0.000***
	Soil type	16.669	1	16.669	35.866	0.000***
	Soil layer	371.839	2	185.92	400.049	0.000***
	Planting pattern * Soil type	13.402	2	6.701	14.419	0.000***
	Planting pattern * Soil layer	8.389	4	2.097	4.513	0.011**
	Soil type * Soil layer	1807.377	2	903.688	1944.494	0.000***
MBP	Intercept	389.068	1	389.068	5390.733	0.000***
	Planting pattern	49.265	2	24.632	341.293	0.000***
	Soil type	2.483	1	2.483	34.399	0.000***
	Soil layer	110.433	2	55.217	765.055	0.000***
	Planting pattern * Soil type	12.879	2	6.439	89.222	0.000***
	Planting pattern * Soil layer	39.217	4	9.804	135.842	0.000***
	Soil type * Soil layer	414.909	2	207.454	2874.382	0.000***
Glu	Intercept	100987.889	1	100987.889	28174.221	0.000***
	Planting pattern	4644.866	2	2322.433	647.927	0.000***
	Soil type	463.487	1	463.487	129.307	0.000***
	Soil layer	22059.803	2	11029.902	3077.19	0.000***
	Planting pattern * Soil type	6486.35	2	3243.175	904.801	0.000***
	Planting pattern * Soil layer	9855.763	4	2463.941	687.405	0.000***
	Soil type * Soil layer	106958.453	2	53479.227	14919.963	0.000***
Ure	Intercept	11997.79	1	11997.79	10807.082	0.000***
	Planting pattern	80.845	2	40.422	36.411	0.000***
	Soil type	1109.051	1	1109.051	998.984	0.000***
	Soil layer	672.444	2	336.222	302.854	0.000***
	Planting pattern * Soil type	34.406	2	17.203	15.496	0.000***
	Planting pattern * Soil layer	12.493	4	3.123	2.813	0.056*
	Soil type * Soil layer	9813.876	2	4906.938	4419.954	0.000***
Acp	Intercept	18909.788	1	18909.788	55929.759	0.000***
	Planting pattern	89.757	2	44.878	132.737	0.000***
	Soil type	1047.011	1	1047.011	3096.76	0.000***
	Soil layer	900.15	2	450.075	1331.193	0.000***
	Planting pattern * Soil type	47.728	2	23.864	70.583	0.000***
	Planting pattern * Soil layer	72.049	4	18.012	53.275	0.000***
	Soil type * Soil layer	16226.545	2	8113.272	23996.745	0.000***
Fe-P	Intercept	81886.714	1	81886.714	2836.874	0.000***
	Planting pattern	11168.857	2	5584.428	193.466	0.000***
	Soil type	483.484	1	483.484	16.75	0.001***
	Soil layer	19607.715	2	9803.858	339.644	0.000***
	Planting pattern * Soil type	4830.924	2	2415.462	83.681	0.000***
	Planting pattern * Soil layer	9723.417	4	2430.854	84.214	0.000***
	Soil type * Soil layer	87078.58	2	43539.29	1508.37	0.000***

	Intercept	2353.976	1	2353.976	1601.619	0.000***
	Planting pattern	175.005	2	87.503	59.536	0.000***
	Soil type	83.307	1	83.307	56.681	0.000***
Al-P	Soil layer	1064.109	2	532.055	362.004	0.000***
	Planting pattern * Soil type	37.496	2	18.748	12.756	0.000***
	Planting pattern * Soil layer	24.139	4	6.035	4.106	0.015**
	Soil type * Soil layer	2708.808	2	1354.404	921.521	0.000***
	Intercept	1343.056	1	1343.056	3622.846	0.000***
	Planting pattern	203.438	2	101.719	274.383	0.000***
	Soil type	18.063	1	18.063	48.724	0.000***
Ca-P	Soil layer	314.539	2	157.27	424.229	0.000***
	Planting pattern * Soil type	234.573	2	117.287	316.377	0.000***
	Planting pattern * Soil layer	358.006	4	89.501	241.427	0.000***
	Soil type * Soil layer	1485.792	2	742.896	2003.936	0.000***
	Intercept	314447.614	1	314447.614	10890.938	0.000***
	Planting pattern	2581.385	2	1290.693	44.703	0.000***
	Soil type	12211.564	1	12211.564	422.949	0.000***
O-P	Soil layer	14022.804	2	7011.402	242.841	0.000***
	Planting pattern * Soil type	109.876	2	54.938	1.903	0.178
	Planting pattern * Soil layer	955.106	4	238.777	8.27	0.001***
	Soil type * Soil layer	278306.51	2	139153.255	4819.593	0.000***
	Intercept	173.395	1	173.395	2773.091	0.000***
	Planting pattern	15.037	2	7.518	120.242	0.000***
	Soil type	0.294	1	0.294	4.7	0.044**
LOP	Soil layer	49.554	2	24.777	396.259	0.000***
	Planting pattern * Soil type	44.113	2	22.056	352.745	0.000***
	Planting pattern * Soil layer	59.397	4	14.849	237.483	0.000***
	Soil type * Soil layer	181.657	2	90.829	1452.611	0.000***
	Intercept	94630.352	1	94630.352	14734.186	0.000***
	Planting pattern	10160.78	2	5080.39	791.03	0.000***
	Soil type	4224.809	1	4224.809	657.813	0.000***
MLOP	Soil layer	6586.786	2	3293.393	512.79	0.000***
	Planting pattern * Soil type	423.876	2	211.938	32.999	0.000***
	Planting pattern * Soil layer	660.792	4	165.198	25.722	0.000***
	Soil type * Soil layer	82641.927	2	41320.963	6433.779	0.000***
	Intercept	19257.758	1	19257.758	4390.175	0.000***
	Planting pattern	2064.132	2	1032.066	235.279	0.000***
	Soil type	292.39	1	292.39	66.656	0.000***
MROP	Soil layer	1237.607	2	618.803	141.068	0.000***
	Planting pattern * Soil type	376.16	2	188.08	42.876	0.000***
	Planting pattern * Soil layer	1509.092	4	377.273	86.007	0.000***
	Soil type * Soil layer	18071.094	2	9035.547	2059.826	0.000***
HROP	Intercept	13084.166	1	13084.166	13796.938	0.000***

Planting pattern	184.245	2	92.123	97.141	0.000***
Soil type	647.932	1	647.932	683.229	0.000***
Soil layer	877.914	2	438.957	462.87	0.000***
Planting pattern * Soil type	393.316	2	196.658	207.371	0.000***
Planting pattern * Soil layer	1695.251	4	423.813	446.9	0.000***
Soil type * Soil layer	11336.6	2	5668.3	5977.086	0.000***

Note：Planting patterns include PP, MPE and MPD. Soil types include rhizosphere soils (R) and bulk soils (B). Soil layers include surface soil (bulk soil 0-20 cm, B1) and subsurface soils (bulk soil 20-40 cm, B2). *,$P < 0.1$; **, $P < 0.05$; ***,$P < 0.01$.
